# Supplementary material for: Diversity and Composition of Fungicolous Fungi Residing in Macrofungi from the Qinling Mountains
Source: J Fungi (Basel). 2024 Aug 25;10(9):601. doi: 10.3390/jof10090601 (PMC11432919; doi:10.3390/jof10090601)
Supplement: Supplementary file 1 [file jof-10-00601-s001.zip › Supplementary figures and tables.pdf]

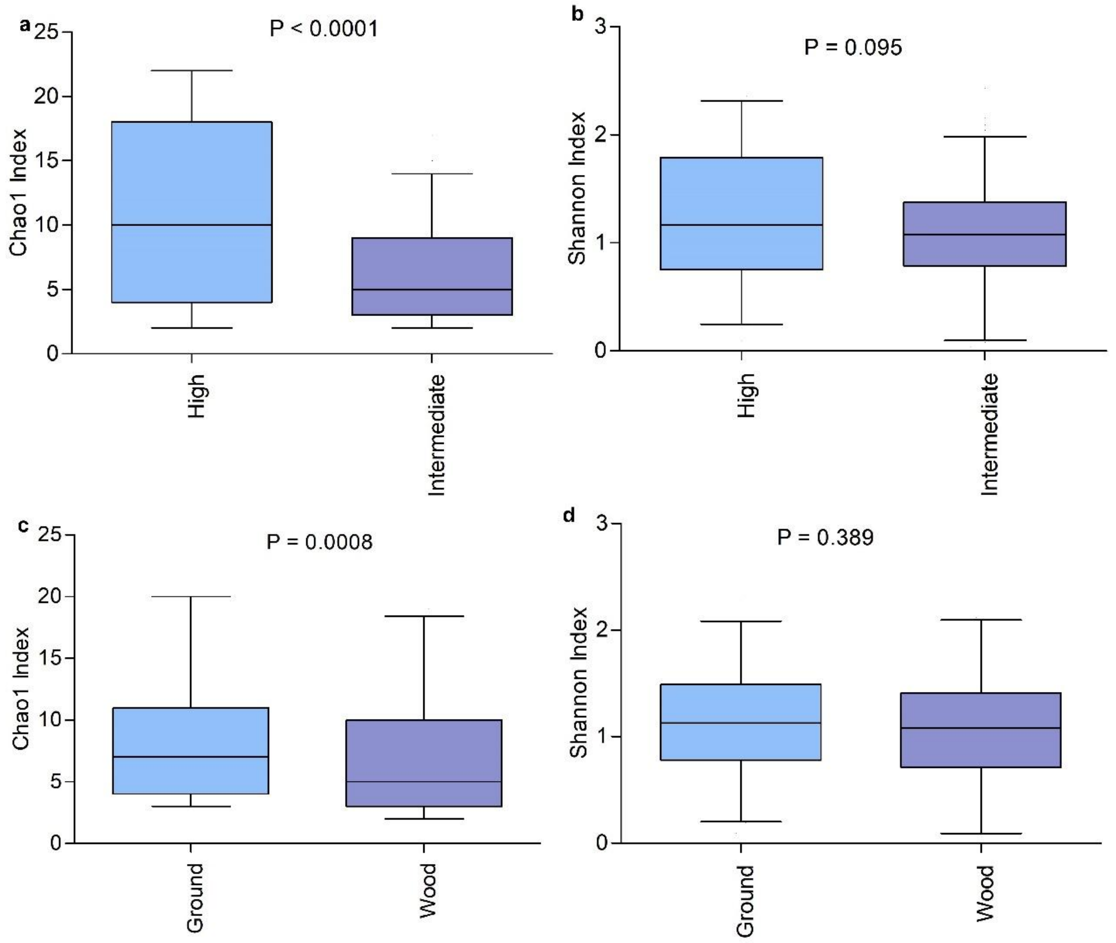

Figure S1. Alpha diversity of fungicolous fungal communities. Box plots show variation in Chao1 (a, c) and Shannon diversity (b, d) indices of fungicolous fungal communities from sporocarps collected from different altitude areas (a, b) and growth substrates (c, d). Medians are indicated by dark lines. Statistical differences between the different categories were evaluated using Student's *t*-tests. Source data are provided as a source data file.

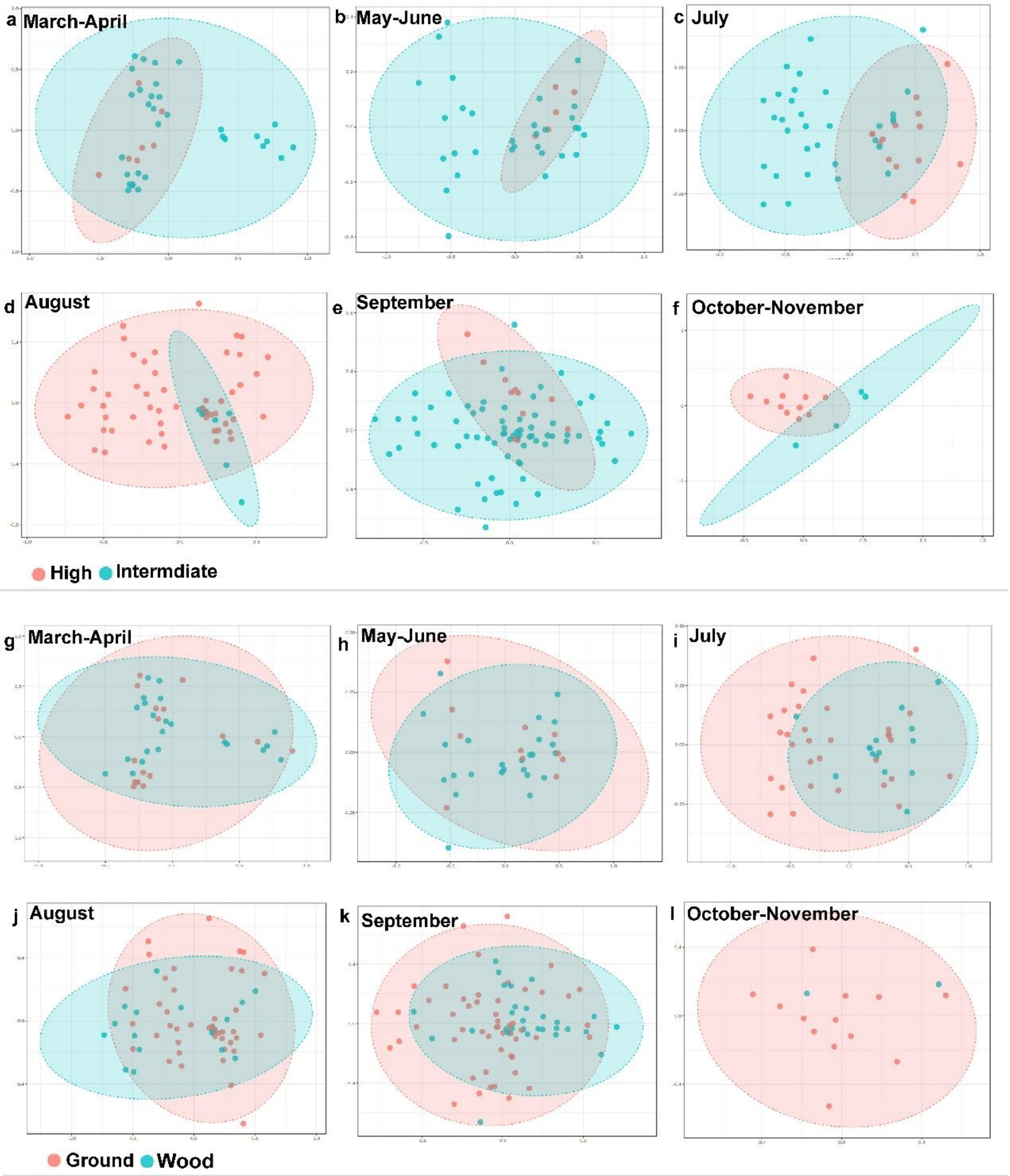

Figure S2. Non-metric multidimensional scaling plots based on Bray–Curtis dissimilarities of fungicolous fungal community composition in sporocarps. These sporocarps were from different altitude areas, growth substrates, and collection times from March to November. Statistical significance was analyzed using PERMANOVA tests. The stress and p values are shown in Table S4. Source data are provided as a source data file.

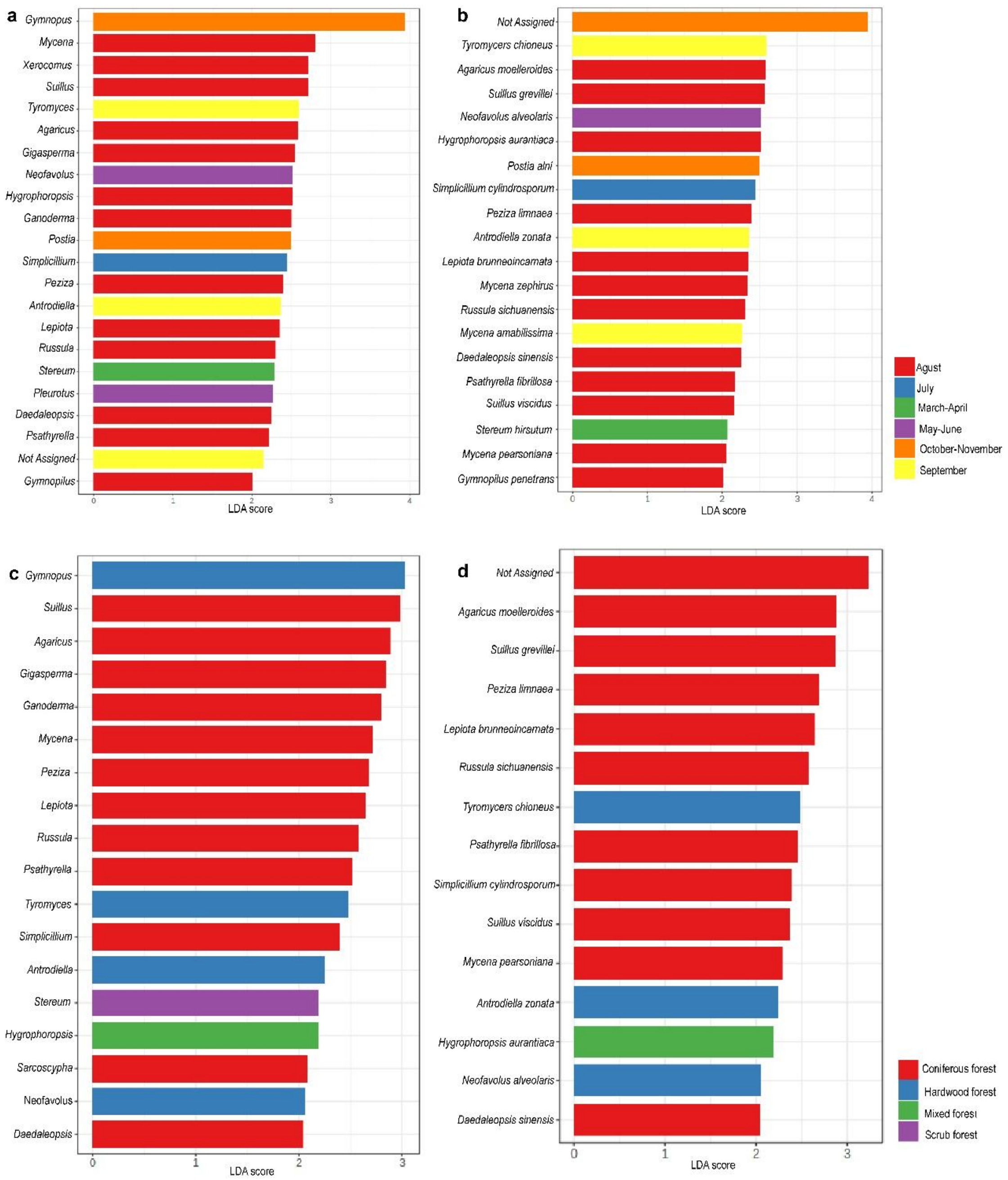

Figure S3. Enrichment of fungicolous fungal taxa in sporocarps. These sporocarps were sampled collected in different months (a and b) and from different habitats (c and d) based on LEfSe analyses. Histograms show linear discriminant analysis (LDA) scores calculated for ASVs at the genus (a and c) and species (b and d) levels. Source data are provided as a source data file.

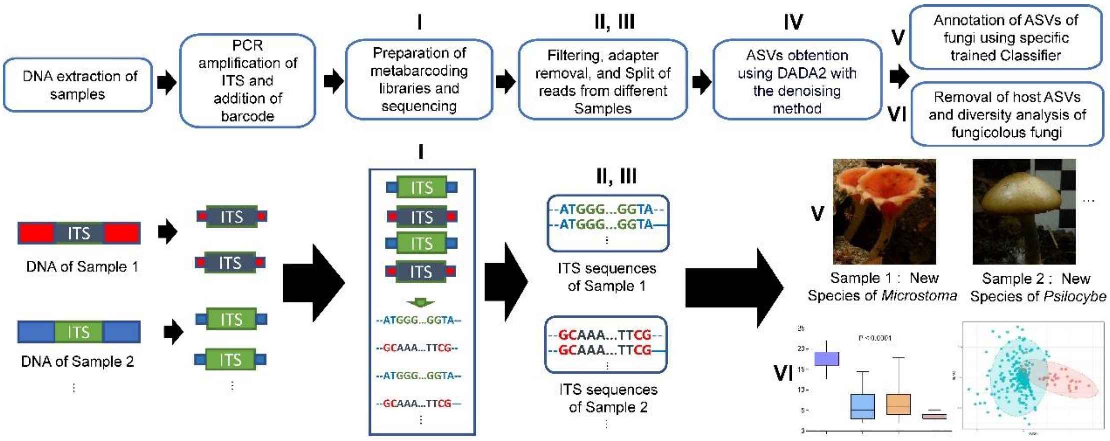

Figure S4. Specialized software pipelines for simultaneous processing of reads from hosts and their associated fungicolous fungi.

Table S1. Novel species identified in this study.

Samples number	Maximum value of percent identity （%）[a]	Notes
20200016	87.72	Published novel species of *Microstoma* 1
20200025	94.10	Published novel species of *Psilocybe* 2
20200053	93.52	Novel species of *Marasmius*
20200118	96.79	Novel species of *Sarcoscypha*
20200143	95.58	Novel species of *Lycoperdon*
20200155	96.09	Novel species of *Russula*
20200161	81.62	Novel species of *Boletaceae*
20200162	95.20	Novel species of *Tricholomopsis*
20200180	91.63	Novel species of *Lactifluus*
20200210	81.96	Published novel species of *Helvella* 3
20200232	81.39	Published novel species of *Helvella* 3
20200244	91.24	Novel species of *Tubaria*
20200273	93.67	Novel species of *Cystolepiota*
20200276	93.89	Novel species of *Geastrum*
20200300	91.87	Novel species of *Inocybe*
20210402	94.14	Novel species of *Psathyrella*
20210441	93.89	Novel species of *Marasmiellus*
20210447	80.81	Novel species of *Conocybe*
20210462	86.64	Novel species of *Inocybe*
20210485	91.87	Novel species of *Cudonia*
20210498	85.61	Novel species of *Ramaria*

[a] Maximum value of percent identity refers to the maximum nucleotide similarity identity value from BLASTn searches using the ITS1 ASV sequences.

References

1. Huo WY et al. MicrostomaNingshanica, a new species of Microstoma based on molecular, light and scanning electron microscopy analyses from Shaanxi Province, China. All Life. 2022;15(1):901-907. DOI: 10.1080/26895293.2022.2114551.
2. He XL, et al. Psilocybe ningshanensis (Hymenogastraceae, Agaricales), a new species from China. Phytotaxa. 2022; 545(2):175-185. DOI: 10.11646/phytotaxa.545.2.6.
3. He XL, et al. Two New Species of Helvella (Pezizales, Ascomycota) in the Qinling Mountains, China. J Fungal Res. 2023.

Table S2. Samples Information

Sample number	Species identification	Notes
20200001	*Psathyrella mammifera*	
20200004	*Psathyrella mammifera*	
20200005	*Psathyrella longicauda*	
20200008	*Tubaria furfuracea*	
20200010	*Sarcoscypha austriaca*	
20200013	*Entoloma vernum*	
20200016	*Microstoma ningshanica*	Published novel species
20200017	*Lentinula edodes*	
20200021	*Sarcoscypha austriaca*	
20200022	*Psathyrella cf. bipellis*	
20200025	*Psilocybe ningshanica*	Published novel species
20200026	*Fulvoderma scaurum*	
20200028	*Psathyrella mammifera*	
20200029	*Psathyrella mammifera*	
20200036	*Neofavolus alveolaris*	
20200037	*Psilocybe ningshanica*	
20200040	*Xylaria sp.*	
20200042	*Mycena galericulata*	
20200043	*Mycena maculate*	
20200044	*Simocybe centunculus*	
20200046	*Scutellinia crinite*	
20200049	*Russula sp.*	
20200051	*Marasmiellus candidus*	
20200052	*Russula sp.*	
20200053	*Marasmius sp.*	Novel species
20200055	*Chaetocalathus galeatus*	
20200056	*Mycena abramsii*	
20200057	*Mycena abramsii*	
20200058	*Laccaria laccata*	
20200063	*Coprinellus radians*	
20200065	*Gymnopus ocior*	
20200067	*Coprinellus disseminatus*	
20200070	*Tyromyces chioneus*	
20200071	*Gymnopus densilamellatus*	
20200072	*Entoloma llimonae*	
20200073	*Gymnopus densilamellatus*	
20200076	*Ganoderma applanatum*	
20200078	*Laccaria laccata*	
20200081	*Russula sp.*	
20200082	*Amanita molliuscula*	
20200083	*Amanita incarnatifolia*	
20200084	*Russula sp.*	

20200085	*Scleroderma cepa*	
20200086	*Tricholomopsis sp.*	
20200087	*Russula sp.*	
20200088	*Entoloma rivulare*	
20200089	*Entoloma rivulare*	
20200090	*Inocybe stellata*	
20200092	*Inocybe malenconii*	
20200093	*Amanita subglobosa*	
20200094	*Russula sp.*	
20200095	*Russula insignis*	
20200096	*Cortinarius epipurrus*	
20200097	*Scleroderma cepa*	
20200098	*Russula sp.*	
20200100	*Russula sp.*	
20200101	*Strobilomyces sp.*	
20200102	*Lactifluus pilosus*	
20200103	*Boletus reticulatus*	
20200105	*Tyromyces kmetii*	
20200114	*Ophiocordyceps nutans*	
20200115	*Trichaleurina tenuispora*	
20200116	*Ganoderma applanatum*	
20200117	*Psathyrella incondita*	
20200118	*Sarcoscypha sp.*	Novel species
20200119	*Polyporus tuberaster*	
20200120	*Psathyrella microrhiza*	
20200121	*Suillus viscidus*	
20200122	*Suillus viscidus*	
20200123	*Psathyrella amarescens*	
20200124	*Tremiscus helvelloides*	
20200125	*Mycena pura*	
20200126	*Mycena pearsoniana*	
20200127	*Suillus grevillea*	
20200128	*Lepiota sp.*	
20200129	*Cystoderma arcticum*	
20200130	*Inocybe cincinnata*	
20200131	*Gymnopilus picreus*	
20200132	*Peziza badia*	
20200134	*Humaria hemisphaerica*	
20200136	*Tephrocybe confusa*	
20200137	*Singerocybe umbilicata*	
20200138	*Tremiscus helvelloides*	
20200139	*Amanita glarea*	
20200140	*Gymnopilus sp.*	
20200141	*Mycena subulate*	

20200142	*Mycena subulate*	
20200143	*Lycoperdon sp.*	Novel species
20200145	*Leotia lubrica*	
20200146	*Tricholomopsis sp.*	
20200147	*Hygrophoropsis ningshanica*	
20200148	*Daedaleopsis confragosa*	
20200151	*Suillus grevillea*	
20200152	*Xerocomus magniporus*	
20200154	*Lactarius deliciosus*	
20200155	*Russula sp.*	Novel species
20200157	*Marasmius occultatiformis*	
20200158	*Marasmius occultatiformis*	
20200159	*Mycena sp.*	
20200161	*Boletaceae sp.*	Novel species
20200162	*Tricholomopsis sp.*	Novel species
20200164	*Strobilomyces sp.*	
20200165	*Gyroporus sp.*	
20200170	*Amanita pallidorosea*	
20200172	*Lactarius ambiguus*	
20200173	*Hymenochaete xerantica*	
20200174	*Suillus luteus*	
20200175	*Amanita griseofolia*	
20200176	*Gymnopus dryophilus*	
20200177	*Hydnellum brunneorubrum*	
20200179	*Hydnum berkeleyanum*	
20200180	*Lactifluus sp.*	Novel species
20200181	*Laetiporus cremeiporus*	
20200182	*Stropharia lignicola*	
20200183	*Laccaria negrimarginata*	
20200184	*Leotia lubrica*	
20200187	*Hydnum albopallidum*	
20200188	*Hydnum albopallidum*	
20200189	*Mycena pura*	
20200191	*Marasmius occultatiformis*	
20200193	*Stropharia rugosoannulata*	
20200197	*Cortinarius scaurus*	
20200199	*Pseudosperma rimosum*	
20200201	*Lactarius subatlanticus*	
20200202	*Mycena pearsoniana*	
20200204	*Mycena galericulata*	
20200205	*Lycoperdon caudatum*	
20200206	*Clavulina sp.*	
20200209	*Inocybe sp.*	
20200210	*Helvella sp.*	Published novel species

20200211	*Hypomyces cervinus*	
20200212	*Hypomyces cervinus*	
20200214	*Polyporus sp.*	
20200215	*Cortinarius helvolus*	
20200216	*Stropharia lignicola*	
20200218	*Singerocybe alboinfundibuliformis*	
20200220	*Mycena citrinomarginata*	
20200222	*Inocybe lilacina*	
20200224	*Mycena floridula*	
20200225	*Lyophyllum leucophaeatum*	
20200226	*Inocybe sp.*	
20200232	*Helvella sp.*	Published novel species
20200234	*Pleurotus pulmonarius*	
20200235	*Psathyrella candolleana*	
20200237	*Pluteus nanus*	
20200240	*Psathyrella microrhiza*	
20200241	*Mucidula mucida*	
20200242	*Coprinopsis lagopus*	
20200243	*Kuehneromyces mutabilis*	
20200244	*Tubaria sp.*	Novel species
20200247	*Cortinarius hinnuleus*	
20200249	*Gymnopus erythropus*	
20200253	*Pleurotus pulmonarius*	
20200256	*Mycena galopus var. candida*	
20200258	*Crepidotus sphaerosporus*	
20200263	*Phillipsia chinensis*	
20200266	*Gymnopus erythropus*	
20200268	*Mycena haematopus*	
20200272	*Antrodiella zonata*	
20200273	*Cystolepiota sp.*	Novel species
20200276	*Geastrum sp.*	Novel species
20200277	*Cyclocybe erebia*	
20200283	*Hypholoma lateritium*	
20200284	*Cyanosporus coeruleivirens*	
20200285	*Agrocybe firma*	
20200290	*Flammulina cephalariae*	
20200292	*Mycena pura*	
20200293	*Suillus luteus*	
20200294	Suillus luteus	
20200295	Leratiomyces sp.	
20200296	*Laccaria laccata*	
20200299	*Cuphophyllus virgineus*	
20200300	*Inocybe sp.*	Novel species

20200301	*Gymnopus dryophilus*
20200302	*Hebeloma cavipes*
20200303	*Hypholoma fasciculare*
20200304	*Laccaria versiforma*
20200323	*Tulostoma squamosum*
20200324	*Geastrum sp.*
20200325	*Hebeloma dunense*
20200326	*Phellinus orientoasiaticus*
20210003	*Tubaria furfuracea*
20210004	*Strobilurus luchuensis*
20210007	*Galerina marginata*
20210009	*Strobilurus luchuensis*
20210011	*Panaeolus papilionaceus*
20210013	*Trametes sanguinea*
20210014	*Tubaria furfuracea*
20210015	*Trametes junipericola*
20210016	*Hypholoma fasciculare*
20210017	*Mycena laevigata*
20210018	*Psathyrella phegophila*
20210019	*Strobilurus luchuensis*
20210020	*Auriscalpium microsporum*
20210026	*Psathyrella phegophila*
20210029	*Neofavolus alveolaris*
20210031	*Galerina marginata*
20210058	*Coprinellus radians*
20210060	*Daedalea dickinsii*
20210062	*Neofavolus alveolaris*
20210063	*Psathyrella senex*
20210064	*Psathyrella longicauda*
20210065	*Psathyrella mammifera*
20210067	*Psathyrella bipellis*
20210068	*Polyporus arcularius*
20210069	*Volvariella gloiocephala*
20210089	*Flammulina cephalariae*
20210090	*Xylaria schweinitzii*
20210091	*Neofavolus alveolaris*
20210092	*Psathyrella amaura*
20210093	*Cerioporus squamosus*
20210094	*Agrocybe praecox*
20210097	*Entoloma sepium*
20210098	*Polyporus varius*
20210099	*Coprinellus radians*
20210100	*Entoloma llimonae*
20210102	*Psathyrella phegophila*

20210104	*Psathyrella bipellis*	
20210106	*Neofavolus alveolaris*	
20210115	*Mycena silvae-nigrae*	
20210118	*Psathyrella candolleana*	
20210121	*Mycena silvae-nigrae*	
20210122	*Turbinellus floccosus*	
20210125	*Artomyces pyxidatus*	
20210128	*Gymnopus dryophilus*	
20210138	*Daedalea dickinsii*	
20210140	*Hydnum berkeleyanum*	
20210141	*Agaricus xanthodermus*	
20210147	*Psathyrella candolleana*	
20210151	*Pluteus primus*	
20210175	*Paxillus involutus*	
20210182	*Strobilomyces confusus*	
20210198	*Russula sp.*	
20210205	*Cortinarius sp.*	
20210208	*Mycena pura*	
20210212	*Russula lakhanpalii*	
20210245	*Geastrum Velutinum*	
20210246	*Gymnopus dryophilus*	
20210251	*Marasmius oreades*	
20210258	*Stropharia ambigua*	
20210261	*Entoloma abortivum*	
20210273	*Trichoglossum sp.*	
20210274	*Lactarius torminosus*	
20210278	*Mucidula mucida*	
20210283	*Phillipsia chinensis*	
20210402	*Psathyrella sp.*	Novel species
20210405	*Polyporus tuberaster*	
20210409	*Coprinellus disseminatus*	
20210411	*Inocybe semifulva*	
20210414	*Boletus reticulatus*	
20210421	*Amanita incarnatifolia*	
20210423	*Humaria hemisphaerica*	
20210426	*Polyporus tuberaster*	
20210427	*Panaeolus fimicola*	
20210433	*Helvella elastica*	
20210434	*Suillus viscidus*	
20210435	*Suillus viscidus*	
20210441	*Marasmiellus sp.*	Novel species
20210445	*Amanita brunneofuliginea*	
20210447	*Conocybe sp.*	Novel species
20210451	*Piptoporellus soloniensis*	

20210453	*Laetiporus cremeiporus*	
20210457	*Amanita pallidorosea*	
20210458	*Russula persicina*	
20210462	*Inocybe sp.*	Novel species
20210465	*Russula aurantioflava*	
20210466	*Gymnopilus penetrans*	
20210468	*Calocera viscosa*	
20210471	*Artomyces pyxidatus*	
20210475	*Hebeloma danicum*	
20210477	*Phillipsia chinensis*	
20210478	*Tricholoma argyraceum*	
20210479	*Lactarius ambiguus*	
20210485	*Cudonia sp.*	Novel species
20210490	*Laccaria laccata*	
20210498	*Ramaria sp.*	Novel species
20210499	*Amanita abrupta*	
20210504	*Suillus luteus*	
20210512	*Armillaria sp.*	
20210525	*Armillaria sp.*	

Table S3. The information of barcodes X

Sample nomber	Barcodes X	
	Forward	Reverse
20200001	ACACGCATGACACACT	AGTGTGTCATGCGTGT
20200004	CATCACTACGCTAGAT	AGTGTGTCATGCGTGT
20200005	TCTATGTCTCAGTAGT	AGTGTGTCATGCGTGT
20200008	GCACATACACGCTCAC	AGTGTGTCATGCGTGT
20200010	TGCTCTCGTGTACTGT	AGTGTGTCATGCGTGT
20200013	GACAGCATCTGCGCTC	AGTGTGTCATGCGTGT
20200016	ATGCTCACTACTACAT	AGTGTGTCATGCGTGT
20200017	GACTGCACATGCACGA	AGTGTGTCATGCGTGT
20200021	CGTGAGTAGTCAGACG	AGTGTGTCATGCGTGT
20200022	ATCGCATCGCAGAGAC	AGTGTGTCATGCGTGT
20200025	TATCAGCACGACATGC	AGTGTGTCATGCGTGT
20200026	ATCGAGCAGCAGTCGT	AGTGTGTCATGCGTGT
20200028	TGCATAGTAGTGCTCT	AGTGTGTCATGCGTGT
20200029	CTACGATGCTATGTAT	AGTGTGTCATGCGTGT
20200036	TACATCTCGCTGCGCA	AGTGTGTCATGCGTGT
20200037	ACACGCATGACACACT	TCTGTAGTGCGTGCGC
20200040	CATCACTACGCTAGAT	TCTGTAGTGCGTGCGC
20200042	GTGCGTATGTCGCTAC	TCTGTAGTGCGTGCGC
20200043	TACGCGTGTACGCAGA	TCTGTAGTGCGTGCGC
20200044	GCACATACACGCTCAC	TCTGTAGTGCGTGCGC
20200046	TGCTCTCGTGTACTGT	TCTGTAGTGCGTGCGC
20200049	GACAGCATCTGCGCTC	TCTGTAGTGCGTGCGC
20200051	CTGATGCGCGCTGTAC	TCTGTAGTGCGTGCGC
20200052	ATGCTCACTACTACAT	TCTGTAGTGCGTGCGC
20200053	GACTGCACATGCACGA	TCTGTAGTGCGTGCGC
20200055	GACGTGTCGTAGATAT	TCTGTAGTGCGTGCGC
20200056	ATCGCTGTGTCTATAG	TCTGTAGTGCGTGCGC
20200057	CGTGAGTAGTCAGACG	TCTGTAGTGCGTGCGC
20200058	ATCGCATCGCAGAGAC	TCTGTAGTGCGTGCGC
20200063	CTAGACACGCAGTCAC	TCTGTAGTGCGTGCGC
20200065	CTACGATGCTATGTAT	TCTGTAGTGCGTGCGC
20200067	TCTACTGCATGATGTC	TCTGTAGTGCGTGCGC
20200070	CATACATCGCGCAGTA	TCTGTAGTGCGTGCGC
20200071	ACACGTGATAGCTACG	TCTGTAGTGCGTGCGC
20200072	TACATCTCGCTGCGCA	TCTGTAGTGCGTGCGC
20200073	ACACGCATGACACACT	AGCTCTGAGTCTCTAT
20200076	CATCACTACGCTAGAT	AGCTCTGAGTCTCTAT
20200078	GTGCGTATGTCGCTAC	AGCTCTGAGTCTCTAT
20200081	ACAGTGCGCTGTCTAT	AGCTCTGAGTCTCTAT
20200082	TGCTCTCGTGTACTGT	AGCTCTGAGTCTCTAT
20200083	CTCAGTGTGACACATG	AGCTCTGAGTCTCTAT

20200084	ATGCTGATGACGCGCT	AGCTCTGAGTCTCTAT
20200085	GACAGCATCTGCGCTC	AGCTCTGAGTCTCTAT
20200086	CGACTACGTACAGTAG	AGCTCTGAGTCTCTAT
20200087	CTGATGCGCGCTGTAC	AGCTCTGAGTCTCTAT
20200088	ATGCTCACTACTACAT	AGCTCTGAGTCTCTAT
20200089	GACTGCACATGCACGA	AGCTCTGAGTCTCTAT
20200090	TATGACTAGTGTACTA	AGCTCTGAGTCTCTAT
20200092	ATCGCTGTGTCTATAG	AGCTCTGAGTCTCTAT
20200093	CGTGAGTAGTCAGACG	AGCTCTGAGTCTCTAT
20200094	ATCGCATCGCAGAGAC	AGCTCTGAGTCTCTAT
20200095	TGCTATCTGAGATACT	AGCTCTGAGTCTCTAT
20200096	CAGCAGATCATGTCGA	AGCTCTGAGTCTCTAT
20200097	TATCAGCACGACATGC	AGCTCTGAGTCTCTAT
20200098	ATCGAGCAGCAGTCGT	AGCTCTGAGTCTCTAT
20200100	TGCATAGTAGTGCTCT	AGCTCTGAGTCTCTAT
20200101	CTACGATGCTATGTAT	AGCTCTGAGTCTCTAT
20200102	GTCACGATATAGTGAC	AGCTCTGAGTCTCTAT
20200103	TCTACTGCATGATGTC	AGCTCTGAGTCTCTAT
20200105	AGTCACACGCACGCTG	AGCTCTGAGTCTCTAT
20200114	GTGCGTATGTCGCTAC	ATCTAGCGTAGTGATG
20200115	TACGCGTGTACGCAGA	ATCTAGCGTAGTGATG
20200116	GCACATACACGCTCAC	ATCTAGCGTAGTGATG
20200117	ACAGTGCGCTGTCTAT	ATCTAGCGTAGTGATG
20200118	TGCTCTCGTGTACTGT	ATCTAGCGTAGTGATG
20200119	CTCAGTGTGACACATG	ATCTAGCGTAGTGATG
20200120	ATGCTGATGACGCGCT	ATCTAGCGTAGTGATG
20200121	GACAGCATCTGCGCTC	ATCTAGCGTAGTGATG
20200122	CGACTACGTACAGTAG	ATCTAGCGTAGTGATG
20200123	CTGATGCGCGCTGTAC	ATCTAGCGTAGTGATG
20200124	ATGCTCACTACTACAT	ATCTAGCGTAGTGATG
20200125	GACTGCACATGCACGA	ATCTAGCGTAGTGATG
20200126	TATGACTAGTGTACTA	ATCTAGCGTAGTGATG
20200127	GACGTGTCGTAGATAT	ATCTAGCGTAGTGATG
20200128	ATCGCTGTGTCTATAG	ATCTAGCGTAGTGATG
20200129	CGTGAGTAGTCAGACG	ATCTAGCGTAGTGATG
20200130	ATCGCATCGCAGAGAC	ATCTAGCGTAGTGATG
20200131	TGCTATCTGAGATACT	ATCTAGCGTAGTGATG
20200132	CAGCAGATCATGTCGA	ATCTAGCGTAGTGATG
20200134	ATCGAGCAGCAGTCGT	ATCTAGCGTAGTGATG
20200136	TGCATAGTAGTGCTCT	ATCTAGCGTAGTGATG
20200137	CTACGATGCTATGTAT	ATCTAGCGTAGTGATG
20200138	GTCACGATATAGTGAC	ATCTAGCGTAGTGATG
20200139	TCTACTGCATGATGTC	ATCTAGCGTAGTGATG
20200140	TAGTGTGCGACTCTGA	ATCTAGCGTAGTGATG

20200141	AGTCACACGCACGCTG	ATCTAGCGTAGTGATG
20200142	CATACATCGCGCAGTA	ATCTAGCGTAGTGATG
20200143	ACACGTGATAGCTACG	ATCTAGCGTAGTGATG
20200145	ACACGCATGACACACT	ACTACTGAGACATAGA
20200146	GCGCACGCACTACAGA	ACTACTGAGACATAGA
20200147	ATAGAGACTCAGAGCT	ACTACTGAGACATAGA
20200148	CATCACTACGCTAGAT	ACTACTGAGACATAGA
20200151	TACGCGTGTACGCAGA	ACTACTGAGACATAGA
20200152	GCACATACACGCTCAC	ACTACTGAGACATAGA
20200154	TGCTCTCGTGTACTGT	ACTACTGAGACATAGA
20200155	CTCAGTGTGACACATG	ACTACTGAGACATAGA
20200157	GACAGCATCTGCGCTC	ACTACTGAGACATAGA
20200158	CGACTACGTACAGTAG	ACTACTGAGACATAGA
20200159	CTGATGCGCGCTGTAC	ACTACTGAGACATAGA
20200161	GACTGCACATGCACGA	ACTACTGAGACATAGA
20200162	TATGACTAGTGTACTA	ACTACTGAGACATAGA
20200164	ATCGCTGTGTCTATAG	ACTACTGAGACATAGA
20200165	CGTGAGTAGTCAGACG	ACTACTGAGACATAGA
20200170	ATCGAGCAGCAGTCGT	ACTACTGAGACATAGA
20200172	TGCATAGTAGTGCTCT	ACTACTGAGACATAGA
20200173	CTACGATGCTATGTAT	ACTACTGAGACATAGA
20200174	GTCACGATATAGTGAC	ACTACTGAGACATAGA
20200175	TCTACTGCATGATGTC	ACTACTGAGACATAGA
20200176	TAGTGTGCGACTCTGA	ACTACTGAGACATAGA
20200177	AGTCACACGCACGCTG	ACTACTGAGACATAGA
20200179	ACACGTGATAGCTACG	ACTACTGAGACATAGA
20200180	TACATCTCGCTGCGCA	ACTACTGAGACATAGA
20200181	ACACGCATGACACACT	GTAGCGACATACGCAC
20200182	GCGCACGCACTACAGA	GTAGCGACATACGCAC
20200183	ATAGAGACTCAGAGCT	GTAGCGACATACGCAC
20200184	CATCACTACGCTAGAT	GTAGCGACATACGCAC
20200187	TACGCGTGTACGCAGA	GTAGCGACATACGCAC
20200188	GCACATACACGCTCAC	GTAGCGACATACGCAC
20200189	ACAGTGCGCTGTCTAT	GTAGCGACATACGCAC
20200191	CTCAGTGTGACACATG	GTAGCGACATACGCAC
20200193	GACAGCATCTGCGCTC	GTAGCGACATACGCAC
20200197	GACTGCACATGCACGA	GTAGCGACATACGCAC
20200199	GACGTGTCGTAGATAT	GTAGCGACATACGCAC
20200201	CGTGAGTAGTCAGACG	GTAGCGACATACGCAC
20200202	ATCGCATCGCAGAGAC	GTAGCGACATACGCAC
20200204	CAGCAGATCATGTCGA	GTAGCGACATACGCAC
20200205	TATCAGCACGACATGC	GTAGCGACATACGCAC
20200206	ATCGAGCAGCAGTCGT	GTAGCGACATACGCAC
20200209	CTACGATGCTATGTAT	GTAGCGACATACGCAC

20200210	GTCACGATATAGTGAC	GTAGCGACATACGCAC
20200211	TCTACTGCATGATGTC	GTAGCGACATACGCAC
20200212	TAGTGTGCGACTCTGA	GTAGCGACATACGCAC
20200214	CATACATCGCGCAGTA	GTAGCGACATACGCAC
20200215	ACACGTGATAGCTACG	GTAGCGACATACGCAC
20200216	TACATCTCGCTGCGCA	GTAGCGACATACGCAC
20200218	GCGCACGCACTACAGA	TCTGCGTACACGCGTA
20200220	CATCACTACGCTAGAT	TCTGCGTACACGCGTA
20200222	GTGCGTATGTCGCTAC	TCTGCGTACACGCGTA
20200224	GCACATACACGCTCAC	TCTGCGTACACGCGTA
20200225	ACAGTGCGCTGTCTAT	TCTGCGTACACGCGTA
20200226	TGCTCTCGTGTACTGT	TCTGCGTACACGCGTA
20200232	ATGCTCACTACTACAT	TCTGCGTACACGCGTA
20200234	TATGACTAGTGTACTA	TCTGCGTACACGCGTA
20200235	GACGTGTCGTAGATAT	TCTGCGTACACGCGTA
20200237	CGTGAGTAGTCAGACG	TCTGCGTACACGCGTA
20200240	CAGCAGATCATGTCGA	TCTGCGTACACGCGTA
20200241	TATCAGCACGACATGC	TCTGCGTACACGCGTA
20200242	ATCGAGCAGCAGTCGT	TCTGCGTACACGCGTA
20200243	CTAGACACGCAGTCAC	TCTGCGTACACGCGTA
20200244	TGCATAGTAGTGCTCT	TCTGCGTACACGCGTA
20200247	TCTACTGCATGATGTC	TCTGCGTACACGCGTA
20200249	AGTCACACGCACGCTG	TCTGCGTACACGCGTA
20200253	ACACGCATGACACACT	GTGAGCGTGTATGTGC
20200256	CATCACTACGCTAGAT	GTGAGCGTGTATGTGC
20200258	GTGCGTATGTCGCTAC	GTGAGCGTGTATGTGC
20200263	CTCAGTGTGACACATG	GTGAGCGTGTATGTGC
20200266	CGACTACGTACAGTAG	GTGAGCGTGTATGTGC
20200268	ATGCTCACTACTACAT	GTGAGCGTGTATGTGC
20200272	ATCGCTGTGTCTATAG	GTGAGCGTGTATGTGC
20200273	CGTGAGTAGTCAGACG	GTGAGCGTGTATGTGC
20200276	CAGCAGATCATGTCGA	GTGAGCGTGTATGTGC
20200277	TATCAGCACGACATGC	GTGAGCGTGTATGTGC
20200283	TCTACTGCATGATGTC	GTGAGCGTGTATGTGC
20200284	TAGTGTGCGACTCTGA	GTGAGCGTGTATGTGC
20200285	AGTCACACGCACGCTG	GTGAGCGTGTATGTGC
20200290	GCGCACGCACTACAGA	ATAGACAGCGCACTGT
20200292	CATCACTACGCTAGAT	ATAGACAGCGCACTGT
20200293	TCTATGTCTCAGTAGT	ATAGACAGCGCACTGT
20200294	GTGCGTATGTCGCTAC	ATAGACAGCGCACTGT
20200295	TACGCGTGTACGCAGA	ATAGACAGCGCACTGT
20200296	GCACATACACGCTCAC	ATAGACAGCGCACTGT
20200299	CTCAGTGTGACACATG	ATAGACAGCGCACTGT
20200300	ATGCTGATGACGCGCT	ATAGACAGCGCACTGT

20200301	GACAGCATCTGCGCTC	ATAGACAGCGCACTGT
20200302	CGACTACGTACAGTAG	ATAGACAGCGCACTGT
20200303	CTGATGCGCGCTGTAC	ATAGACAGCGCACTGT
20200304	ATGCTCACTACTACAT	ATAGACAGCGCACTGT
20200323	ACACGTGATAGCTACG	ATAGACAGCGCACTGT
20200324	TACATCTCGCTGCGCA	ATAGACAGCGCACTGT
20200325	ACACGCATGACACACT	ACAGTACACGAGAGCA
20200326	GCGCACGCACTACAGA	ACAGTACACGAGAGCA
20210003	TCTATGTCTCAGTAGT	ACAGTACACGAGAGCA
20210004	GTGCGTATGTCGCTAC	ACAGTACACGAGAGCA
20210007	ACAGTGCGCTGTCTAT	ACAGTACACGAGAGCA
20210009	CTCAGTGTGACACATG	ACAGTACACGAGAGCA
20210011	GACAGCATCTGCGCTC	ACAGTACACGAGAGCA
20210013	CTGATGCGCGCTGTAC	ACAGTACACGAGAGCA
20210014	ATGCTCACTACTACAT	ACAGTACACGAGAGCA
20210015	GACTGCACATGCACGA	ACAGTACACGAGAGCA
20210016	TATGACTAGTGTACTA	ACAGTACACGAGAGCA
20210017	GACGTGTCGTAGATAT	ACAGTACACGAGAGCA
20210018	ATCGCTGTGTCTATAG	ACAGTACACGAGAGCA
20210019	CGTGAGTAGTCAGACG	ACAGTACACGAGAGCA
20210020	ATCGCATCGCAGAGAC	ACAGTACACGAGAGCA
20210026	TGCATAGTAGTGCTCT	ACAGTACACGAGAGCA
20210029	TCTACTGCATGATGTC	ACAGTACACGAGAGCA
20210031	AGTCACACGCACGCTG	ACAGTACACGAGAGCA
20210058	CAGCAGATCATGTCGA	CATGTGTCACACTGAG
20210060	ATCGAGCAGCAGTCGT	CATGTGTCACACTGAG
20210062	TGCATAGTAGTGCTCT	CATGTGTCACACTGAG
20210063	CTACGATGCTATGTAT	CATGTGTCACACTGAG
20210064	GTCACGATATAGTGAC	CATGTGTCACACTGAG
20210065	TCTACTGCATGATGTC	CATGTGTCACACTGAG
20210067	AGTCACACGCACGCTG	CATGTGTCACACTGAG
20210068	CATACATCGCGCAGTA	CATGTGTCACACTGAG
20210069	ACACGTGATAGCTACG	CATGTGTCACACTGAG
20210089	GACGTGTCGTAGATAT	AGCGCGTCATCAGCAT
20210090	ATCGCTGTGTCTATAG	AGCGCGTCATCAGCAT
20210091	CGTGAGTAGTCAGACG	AGCGCGTCATCAGCAT
20210092	ATCGCATCGCAGAGAC	AGCGCGTCATCAGCAT
20210093	TGCTATCTGAGATACT	AGCGCGTCATCAGCAT
20210094	CAGCAGATCATGTCGA	AGCGCGTCATCAGCAT
20210097	CTAGACACGCAGTCAC	AGCGCGTCATCAGCAT
20210098	TGCATAGTAGTGCTCT	AGCGCGTCATCAGCAT
20210099	CTACGATGCTATGTAT	AGCGCGTCATCAGCAT
20210100	GTCACGATATAGTGAC	AGCGCGTCATCAGCAT
20210102	TAGTGTGCGACTCTGA	AGCGCGTCATCAGCAT

20210104	CATACATCGCGCAGTA	AGCGCGTCATCAGCAT
20210106	TACATCTCGCTGCGCA	AGCGCGTCATCAGCAT
20210115	ACACGCATGACACACT	AGTGTGTCATGCGTGT
20210118	ATAGAGACTCAGAGCT	AGCTCTGAGTCTCTAT
20210121	CATCACTACGCTAGAT	ATCTAGCGTAGTGATG
20210122	TCTATGTCTCAGTAGT	ACTACTGAGACATAGA
20210125	GTGCGTATGTCGCTAC	GTAGCGACATACGCAC
20210128	TACGCGTGTACGCAGA	TCTGCGTACACGCGTA
20210138	CTCAGTGTGACACATG	CATGTGTCACACTGAG
20210140	ATGCTGATGACGCGCT	AGCGCGTCATCAGCAT
20210141	GACAGCATCTGCGCTC	GAGCGCAGATGCTGTC
20210147	GACTGCACATGCACGA	TCGTGCATGTGCAGTC
20210151	TATGACTAGTGTACTA	TAGTACACTAGTCATA
20210175	ATCGAGCAGCAGTCGT	ACGACTGCTGCTCGAT
20210182	TGCATAGTAGTGCTCT	AGAGCACTACTATGCA
20210198	CATACATCGCGCAGTA	TACTGCGCGATGTATG
20210205	TACATCTCGCTGCGCA	TGCGCAGCGAGATGTA
20210208	ACACGCATGACACACT	AGTGTGTCATGCGTGT
20210212	GCGCACGCACTACAGA	TCTGTAGTGCGTGCGC
20210245	GACAGCATCTGCGCTC	GAGCGCAGATGCTGTC
20210246	CGACTACGTACAGTAG	CTACTGTACGTAGTCG
20210251	ATGCTCACTACTACAT	ATGTAGTAGTGAGCAT
20210258	GACTGCACATGCACGA	TCGTGCATGTGCAGTC
20210261	TATGACTAGTGTACTA	TAGTACACTAGTCATA
20210273	GACGTGTCGTAGATAT	ATATCTACGACACGTC
20210274	ATCGCTGTGTCTATAG	CTATAGACACAGCGAT
20210278	TGCTATCTGAGATACT	AGTATCTCAGATAGCA
20210283	ATCGAGCAGCAGTCGT	ACGACTGCTGCTCGAT
20210402	TGCATAGTAGTGCTCT	AGAGCACTACTATGCA
20210405	CTACGATGCTATGTAT	ATACATAGCATCGTAG
20210409	GTCACGATATAGTGAC	GTCACTATATCGTGAC
20210411	TCTACTGCATGATGTC	GACATCATGCAGTAGA
20210414	CATACATCGCGCAGTA	TACTGCGCGATGTATG
20210421	TCTATGTCTCAGTAGT	ACTACTGAGACATAGA
20210423	TACGCGTGTACGCAGA	TCTGCGTACACGCGTA
20210426	TGCTCTCGTGTACTGT	ACAGTACACGAGAGCA
20210427	CTCAGTGTGACACATG	CATGTGTCACACTGAG
20210433	ATGCTCACTACTACAT	ATGTAGTAGTGAGCAT
20210434	GACTGCACATGCACGA	TCGTGCATGTGCAGTC
20210435	TATGACTAGTGTACTA	TAGTACACTAGTCATA
20210441	CGTGAGTAGTCAGACG	CGTCTGACTACTCACG
20210445	TGCTATCTGAGATACT	AGTATCTCAGATAGCA
20210447	CAGCAGATCATGTCGA	TCGACATGATCTGCTG
20210451	TATCAGCACGACATGC	GCATGTCGTGCTGATA

20210453	ATCGAGCAGCAGTCGT	ACGACTGCTGCTCGAT
20210457	CTACGATGCTATGTAT	ATACATAGCATCGTAG
20210458	GTCACGATATAGTGAC	GTCACTATATCGTGAC
20210462	TAGTGTGCGACTCTGA	TCAGAGTCGCACACTA
20210465	AGTCACACGCACGCTG	CAGCGTGCGTGTGACT
20210466	CATACATCGCGCAGTA	TACTGCGCGATGTATG
20210468	ACACGTGATAGCTACG	CGTAGCTATCACGTGT
20210471	ACACGCATGACACACT	AGTGTGTCATGCGTGT
20210475	GCGCACGCACTACAGA	TCTGTAGTGCGTGCGC
20210477	ATAGAGACTCAGAGCT	AGCTCTGAGTCTCTAT
20210478	CATCACTACGCTAGAT	ATCTAGCGTAGTGATG
20210479	TCTATGTCTCAGTAGT	ACTACTGAGACATAGA
20210485	TACGCGTGTACGCAGA	TCTGCGTACACGCGTA
20210490	ACAGTGCGCTGTCTAT	ATAGACAGCGCACTGT
20210498	TGCTCTCGTGTACTGT	ACAGTACACGAGAGCA
20210499	CTCAGTGTGACACATG	CATGTGTCACACTGAG
20210504	GACAGCATCTGCGCTC	GAGCGCAGATGCTGTC
20210512	CGACTACGTACAGTAG	CTACTGTACGTAGTCG
20210525	GACTGCACATGCACGA	TCGTGCATGTGCAGTC

Table S4. The stresses and p values of NMDS plots of Figure S2.

NMDS plot	Stress	p values
a	0.08009	0.039
b	0.087392	0.059
c	0.09035	0.001
d	0.14873	0.001
e	0.18674	0.037
f	0.1295	0.001
g	0.08009	0.063
h	0.087392	0.648
i	0.09035	0.001
j	0.14873	0.027
k	0.18674	0.001
l	0.1295	0.111

Table S5. Trophic modes of host species.

Trophic modes	Number of host species		
	**Grown on ground**	**Grown on wood**	**Total**
Saprotroph	68	79	147
Pathotroph	1	1	2
Symbiotroph	70	5	75
Others	30	26	56

"Grown on ground" specifically means "on the forest floor". source data are provided as a source data file.
